# Barriers to the use of tests for early detection of colorectal cancer in Chile

**DOI:** 10.1038/s41598-024-58920-z

**Published:** 2024-04-17

**Authors:** Gabriela Alfaro, Zoltan Berger, Susana Mondschein, Felipe Subiabre, Natalia Yankovic

**Affiliations:** 1 Industrial Engineering Department, University of Chile, Beauchef 851, Santiago, Chile; 2 Department of Medicine, Gastroenterology Section, Hospital Clínico de La Universidad de Chile, Santiago, Chile; 3 Complex Engineering Systems Institute, Santiago, Chile; 4 Center for Cancer Prevention and Control (CECAN), Santiago, Chile; 5 ESE Business School, Universidad de los Andes, Santiago, Chile

**Keywords:** Colorectal cancer, Chile, Colonoscopies, Barriers to colon cancer screening, Cancer prevention, Cancer, Health care

## Abstract

This study aimed to assess the use of colorectal cancer (CRC) tests for prevention and early detection, alongside exploring the associated barriers to these tests. A stratified national survey was conducted in Chile, involving 1893 respondents (with a 2.3% error margin and 95% confidence interval). Logistic and multinomial regression analyses were employed to examine variations in test utilization likelihood and barrier. We found that the key determinants for undergoing CRC tests included age, health status, possession of private health insurance, and attainment of postgraduate education. Notably, 18% and 29% of respondents covered by public and private insurance, respectively, cited personal prevention as the primary motivation for test uptake. The principal obstacle identified was lack of knowledge, mentioned by 65% of respondents, while 29% and 19% of the publicly and privately insured respectively highlighted lack of access as a barrier. The results of this study provide valuable insights into factors influencing CRC screening, aiming to inform public health policies for expanding national coverage beyond diagnosis and treatment to encompass preventive measures.

## Introduction

Colorectal cancer (CRC) is a significant health issue globally, representing over 10% of all cancer diagnoses^1^. Incidence rates vary geographically, with higher rates in developed countries^2,3^. By 2035, 2.5 million new cases of CRC will be diagnosed worldwide^4^. A lifestyle-related disease, CRC can be prevented by changes in habits, such as maintaining a healthy diet, physical activity, and avoiding smoking and alcohol^5^. CRC can also be prevented by the detection and removal of adenomas, which can be done during a colonoscopy, which accounts for more prevention in the U.S. in the most recent period than modifying lifestyles^6^.

The Chilean health system is a hybrid of public and private health providers and insurers^7^. Patients with private insurance (ISAPRE, 14.4% of the population) can only access private providers, while those with public insurance (FONASA, 78% of the population) can access public and private providers. Families of military and police forces have special health insurance and providers (Armed Forces, 2.8% of the population). Although the most recent guidelines recommend CRC screening among average-risk adults between 45 and 75 years of age^8^, in Chile, there are no systematic policies for the early detection of CRC, nor there exist any incentives to doctors recommending (or not recommending) such screening. In this way, patients with ISAPRE can get access to screening with coverages and copayments that will depend on their individual plans. Those with FONASA insurance, can access private providers for screening with a much higher copayment and with very limited availability for colonoscopies. For the Armed Forces insurance, there exists not only special providers, but also specific policies, and procedures, especially concerning preventive medicine and the existence of screening protocols. In the supplementary material, “Appendix A”, we describe FONASA groups including access and coverages under the public providers.

CRC treatment is part of the Explicit Guarantees in Health (GES) plan, which guarantees access, opportunity, and financial protection for all Chilean citizens regardless of their health insurance for a set of conditions and diseases.

In recent years, there has been an increase in both incidence and mortality from CRC in Chile^9^. Our previous research found important inequalities in the survival probabilities of patients with CRC in Chile, with the 5-year survival probability varying depending on the type of health insurance, with a probability of 64% for ISAPRE patients and only 31% for those affiliated with FONASA group with lower income. The geographic location of the patient played a statistically significant role in the 5-year probability of survival^9^. Differences in the detection barriers of CRC have been reported in the literature, depending on sociodemographic factors, access to diagnostic tests, and the general state of health of the patient^10,11^.

The study of whether there are differences in the detection barriers for CRC is of particular interest since, depending on the level of prevention and early detection, the chances of survival vary. The diagnosis of CRC requires colonoscopy and biopsy, definitive endoscopic surgery in the initial stages, which can also be performed in the same procedure which transforms the diagnostic procedure into a therapeutic one^6^. The PRENEC program is a positive experience published in Chile, a study of 24,285 asymptomatic individuals between 50 and 75 years of age^12^. It found that of these, 3623 (15.1%) individuals had a positive fecal immunochemical test (FIT) result. 202 of them had cancer, and precancerous adenomatous polyps were found in an additional 1853 individuals. Worldwide, most experts accept that colonoscopy reduces the incidence and mortality of CRC^13^, but high-sensitive FIT tests can also detect cancer and advance polyps and are a good method to select patients for colonoscopy^14^.

In Chile, the prevalence of colonoscopy in adults between 50 and 75 years was estimated at 8.7% in 2009–2010, using information from the national health survey^15^.

This work is a first attempt to identify the causes of the differences in the survival probabilities of CRC for the Chilean population, exploring, through a national survey, the barriers to the use of CRC screening options. Using the information collected from the survey we also estimate the annual number of colonoscopies currently performed in the country.

## Methods

### Study population

The study conducted a national survey in Chile using a panel stratified by gender, age, geographic location (north, metropolitan region (RM), and south), and socio-economic status (SES). There are seven categories for SES according to household income and size (Table E.1 of Appendix B in the supplementary material for detailed description).

The survey included respondents aged 45 years and above, with a final sample size of 1812 after exclusions due to incomplete surveys and a history of CRC to avoid overestimating the prevalence of screening it was conducted electronically between August and September 2022, with a 2.3% error at a 95% confidence interval. The survey aimed to provide information on awareness, knowledge, and attitudes towards CRC screening in Chile. The company Netquest provided the panel of respondents, and the questionnaire was coded in Qualtrics^16^. The study aimed to understand the factors that may affect the uptake of CRC screening in Chile, given that medical recommendations for screening colonoscopies are performed anecdotally and depend on the criteria of each professional.

Table 1 summarizes the population in the sample by groups, including the weight of the group in the overall Chilean population using the information from the 2017 census^17^.

**Table 1:** Percentage of the national population corresponding to each age group, sex, and geographic location, and the number of samples in the survey (I = number of incomplete surveys, CRC = number of respondents with a history of CRC).

Region	Sex	Age group	People in the population		People in the sample	
			Number	(%)	Number	(I, CRC)
RM	Men	45–54	440,937	2.5	214	(5, 0)
		54+	670,224	3.8	176	(4, 1)
	Female	45–54	493,849	2.8	243	(6, 1)
		54+	846,599	4.8	292	(8, 3)
North–South	Men	45–54	687,862	3.9	271	(10, 0)
		54+	1,111,161	6.3	285	(13, 10)
	Female	45–54	723,137	4.1	223	(14, 0)
		54+	1,269,898	7.2	189	(5, 1)
Total			6,243,667	35.4	1893	(65, 16)

**Figure 1:**
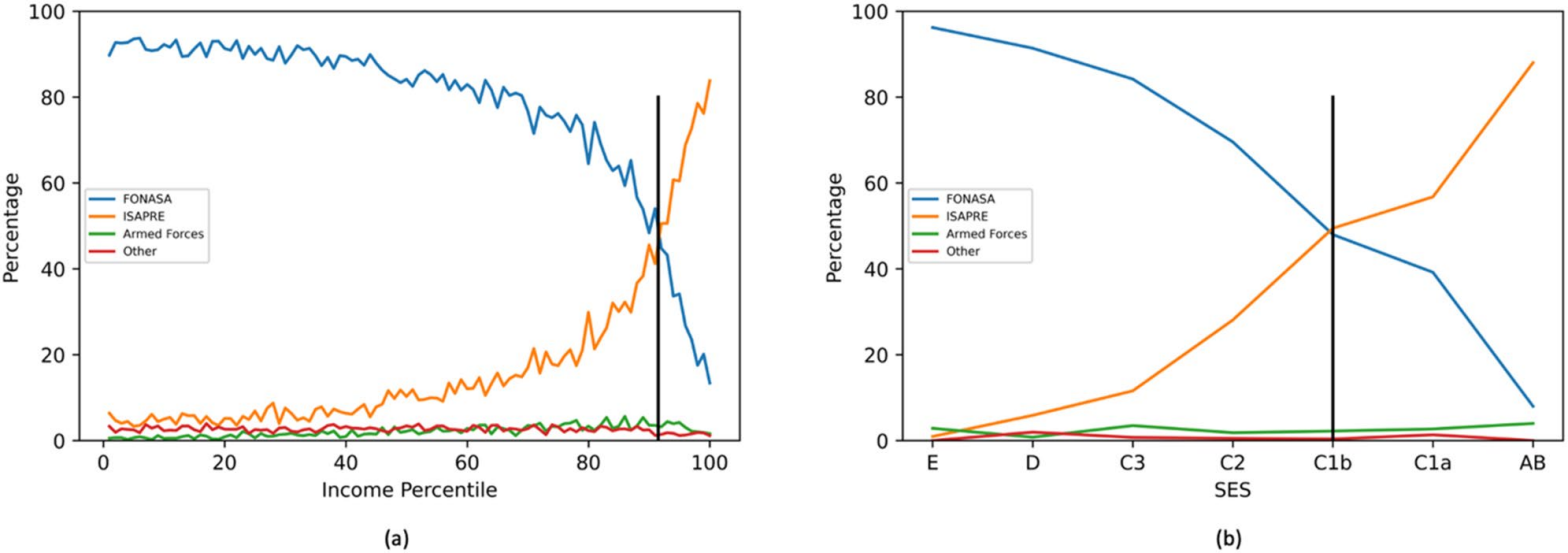
Percentage of population with specific health insurance according to their income or SES. (**a**) Shows the overall population with data from ^7^. (**b**) The distribution of people in the sample.

In Chile, there is a clear correlation between the SES and the type of insurance of the population. This is true not only within FONASA groups but also for the privately insured in ISAPRE. Figure 1a shows the proportion of the total population insured in the FONASA and ISAPRE depending on their income level. It is worth noticing that in the overall population at the 91st income percentile we have 50% of the population insured in ISAPRE and FONASA respectively, corresponding to $742.778 monthly per capita CLP. Figure 1b presents the information from the survey, where income levels have been replaced by SES, with 50% of the sample in each category corresponding to SES C1b, with a monthly per capita income between $662.000 and $1.100.000, consistent with the information from the overall population.

The distribution of SES for the population insured by the police and armed is between ISAPRE and FONASA. 2.3% of the sample had this special health system, in line with the 2.8% for the total population.

### Measurements

Respondents reported if they have had a screening exam for CRC, and for those with an affirmative answer how long ago they have had the exam and the reasons why they had undergone it. For the participants who had never undergone this type of examination, we sought to understand the barriers to the use of CRC detection tests.

We collected information from sociodemographic covariates that were available in the panel data: age, sex, educational level, SES, and geographical location. Respondents reported whether they had a history of CRC, their knowledge of CRC, health insurance, the time elapsed since their last medical check-up, and their general their state of health. “Appendix C” in the supplementary material presents the description of the survey.

### Statistical analysis

#### Grouping of variables

The reasons for obtaining preventive examinations were categorized into four groups: symptoms, medical prevention, personal prevention, and others. The category “symptoms” allows the inclusion of patients offered with a diagnostic test, which may be considered as a preventive or screening test by the person participating in the survey. For people without symptoms, the trigger for requesting the test may have been the doctor (medical prevention) or the patient's personal motivation (personal prevention) or some other reasons (“I have a different medical condition that requires a colonoscopy”). In the same way, barriers to obtaining CRC examinations were categorized into the following groups: lack of access (lack of time, exams are expensive or there is no availability in the region), psychosocial barriers (scared or dislike the exam, forget to do it), lack of knowledge (I don’t know them, I don’t need them, I’m not old enough, I don’t know where to get tested), and others. Any free text entered by the respondents was read and classified into one of the categories described above (for instance “I only get access if I have symptoms” or “there are not appointments available” were classified as lack of access), leaving the motivations that could not be classified in the category "Other" (for instance “I don’t have symptoms” or “I’m healthy”).

Respondents who expressed various reasons or barriers to obtaining CRC screening were assigned to a single category as detailed in “Appendix D” in the supplementary material.

#### Information analysis

We present the descriptive analysis of the survey results, according to the available sociodemographic information, as well as the frequency of reasons (barriers) to obtain (to prevent) CRC exams. We explore any difference in reasons (barriers) considering individuals’ health insurance, since in Chile there are well-known differences in access to and coverage for exams and specialist care based on health insurance. Using the percentages of people who have had colonoscopies according to age groups and SES we can extrapolate the results of the survey to the total population.

We use a logistic regression model to evaluate the variables affecting CRC screening. The variables considered in the model included type of health insurance, educational level, sex, SES, age, geographic region, self-reported health status, and time since the last health check-up. The specification of the model was chosen using the criterion of the lowest Akaike concordance index (AIC)^18^.

The study also sought differences in the sociodemographic characteristics of respondents who did not undergo CRC diagnostic tests due to various barriers, using a multinomial logistic regression model. The study's findings could help inform strategies to improve CRC screening rates and reduce health disparities in Chile.

### Ethics declarations

The project has institutional approval from the Ethics and Biosafety Committee for Research from the Facultad de Ciencias Físicas y Matemáticas (FCFM) University of Chile. All methods were performed in accordance with the relevant guidelines and regulations. Informed consent was obtained from all participants.

## Results

### Descriptive analysis

Table 2 presents the characteristics of the sample, including their use of CRC screening or diagnostic tests, based on sociodemographic factors, health insurance, knowledge of CRC, medical check-ups, and self-perception of health status. Among the total sample, 20.3% reported having undergone CRC tests, with a higher percentage for women, older individuals, those with higher education and higher socioeconomic status, and those with health insurance from the Armed Forces or ISAPRE. Those with more knowledge about CRC and those who had recent medical check-ups were also more likely to have undergone screening or diagnostic tests.

**Table 2:** CRC screening by sociodemographic characteristics (1: Colonoscopy, 2: FIT, 3: Both tests).

		*Have you been screened for CRC?*					
					1	2	3
		No	(%)	Yes	(%)	(%)	(%)
Age							
45–49	476	401	84.2	75	9.0	3.6	3.2
50–54	439	373	85.0	66	8.7	3.0	3.4
55–59	297	248	83.5	49	10.4	2.4	3.7
60–64	239	173	72.4	66	13.4	6.7	7.5
64–69	202	144	71.3	58	20.8	4.5	3.5
70+	159	105	66.0	54	22.6	5.0	6.3
Sex							
Male	903	730	80.8	173	9.9	4.3	5.0
Female	909	714	78.5	195	14.5	3.4	3.5
Education							
Compulsory education	548	466	85.0	82	8.9	3.1	2.9
Senior technician	589	467	79.3	122	12.2	4.6	3.9
Professional	498	388	77.9	110	13.3	4.4	4.4
Postgraduate	177	123	69.5	54	19.2	2.3	9.0
SES							
AB	49	35	71.4	14	20.4	0.0	8.2
C1a	221	161	72.9	60	18.6	3.6	5.0
C1b	266	202	75.9	64	16.2	1.9	6.0
C2	376	291	77.4	85	11.2	6.6	4.8
C3	541	452	83.5	89	8.3	4.1	4.1
D	255	215	84.3	40	12.2	2.4	1.2
E	104	88	84.6	16	8.7	3.8	2.9
Health insurance							
ISAPRE	486	342	70.4	144	20.6	3.3	5.8
FONASA groups C and D	605	501	82.8	104	8.6	5.1	3.5
FONASA groups A and B	491	413	84.1	78	9.2	3.5	3.3
FONASA does not know group	173	148	85.5	25	9.2	1.2	4.0
Armed Forces	43	27	62.8	16	18.6	7.0	11.6
None/Other	14	13	92.9	1	0.0	7.1	0.0
Region							
North	416	343	82.5	73	7.7	5.8	4.1
RM	897	702	78.3	195	13.3	4.0	4.5
South	499	399	80.0	100	14.0	2.0	4.0
Knowledge of CRC							
Low	237	216	91.1	21	5.5	2.1	1.3
Medium low	1082	905	83.6	175	9.3	3.6	3.2
Medium high	273	198	72.5	74	14.7	6.6	5.9
High	99	56	56.6	43	30.3	4.0	9.1
Very high	121	66	54.5	55	30.6	3.3	11.6
Last medical check-up							
Less than a year ago	1075	796	74.0	279	16.0	4.7	5.3
Between 1 and 3 years	397	332	83.6	65	8.1	4.3	3.8
More than 3 years ago	188	170	90.4	18	5.9	1.1	2.7
I don’t remember	152	146	96.1	6	3.9	0.7	0.0
Perception of health status							
Very bad	22	16	72.7	6	13.6	9.1	4.5
Bad	65	49	75.4	16	15.4	4.6	4.6
Fair	557	443	79.5	114	12.4	3.9	4.1
Good	992	794	80.0	198	12.3	3.7	3.9
Excellent	176	142	80.7	34	9.7	3.4	6.3
TOTAL	1.81	1444	79.6	368	12.3	3.9	4.2

**Figure 2:**
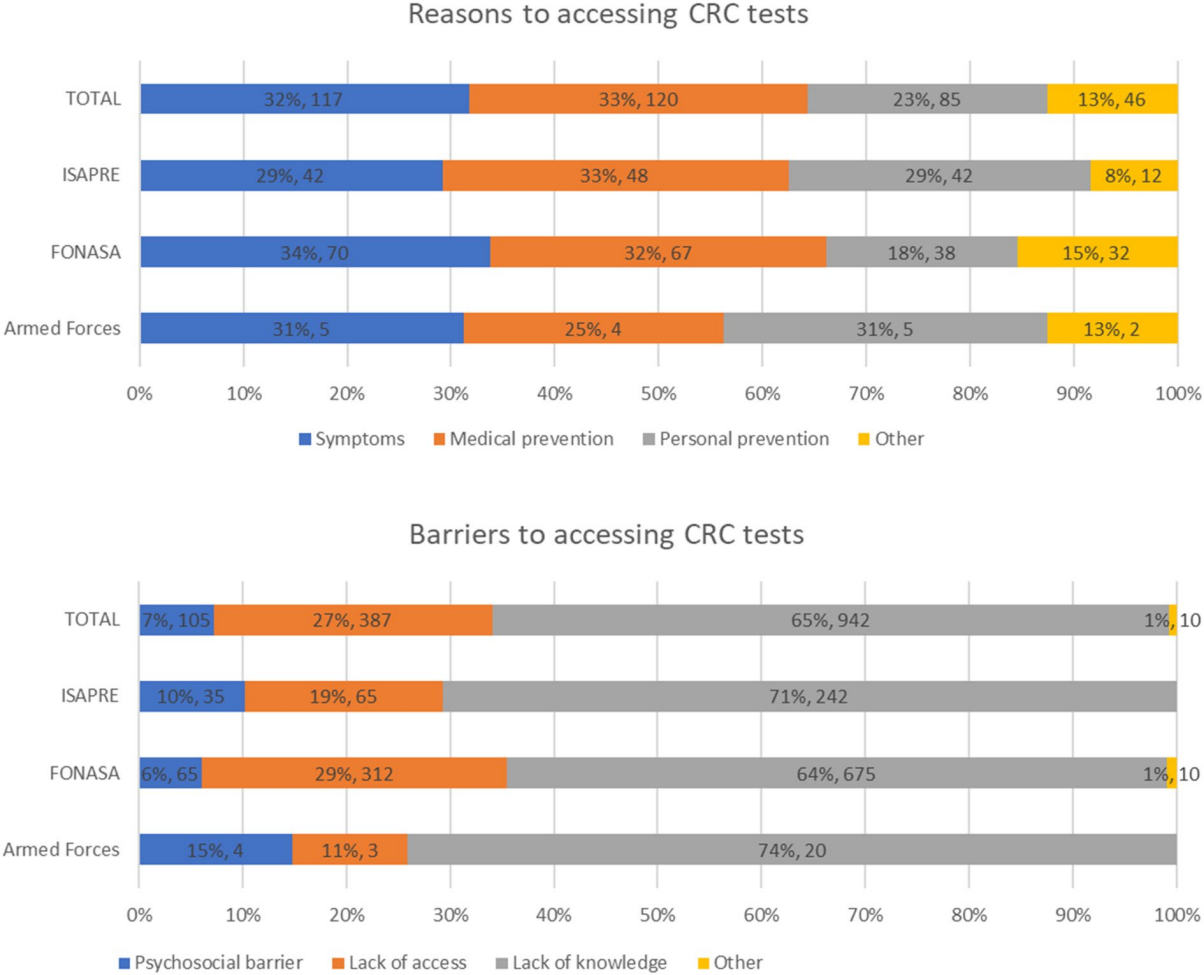
Reasons and barriers to perform CRC screening and diagnostic tests considering health insurance (percentage and number of people in the sample). Total includes 14 people with other or no health insurance.

For those who had undergone CRC tests, the main reasons were doctor’s recommendation and their own symptoms. Among those who had not undergone CRC tests, the most prevalent barrier was ignorance, followed by lack of access, with a small percentage citing psychosocial barriers. The barriers varied by health insurance, with FONASA respondents more likely to cite lack of access and less likely to cite psychosocial barriers compared to those with ISAPRE or Armed Forces health insurance. Figure 2 shows the reasons and barriers to performing CRC tests for different health insurance. We have observed some differences in the mentioned reasons and barriers between men and women, with a larger fraction of women accessing CRC test due the presence of symptoms (36.9% for women vs. 26% for men) and a larger fraction of women mentioning lack of knowledge as the main barrier to perform a CRC test (69.1% of women vs. 61.1% of men). Table E.1 in the Appendix E of the supplementary material contains the full description considering the different health insurance.

Using the percentages of people who have had colonoscopies according to age groups and SES from Table 2 we can extrapolate the results of the survey to the total population reported in Table 1. It can be concluded that among those over 45 years, around 1,083,256 people would have ever had preventive or diagnostic colonoscopies. This corresponds to 17.3% of the population over 45 years of age, slightly higher than the in-sample percentage of 16.5%. Moreover, 62.8% of the respondents reported having done so in the past 5 years, corresponding to 680,319 colonoscopies in that period, which would correspond to 136,034 procedures per year.

The survey included a question about how long ago the latest CRC screening was performed. This may shed some light on how the testing has evolved in the last years. Table 3 presents the information from the survey, including the type of screening and people “not remembering” how long ago the screening was performed. The main changes are an increasing proportion of screening in the later years (66% of people undergoing screening reported to have done it in the past 5 years) and an even larger increase in the number of people undergoing FIT screening (70% of all FIT tests were performed in the last 5 years), which may be due an increase on awareness about CRC and the existence of screening tests. 36 individuals (9.8% of the total) did not remember when the testing was performed.

**Table 3:** Evolution of CRC screening over time, considering the 368 individuals who answered they have never had a CRC screening test.

	More than 10 years	Between 5 and 10 years	Less than 5 years	I don't remember	Total
Colonoscopy	14	59	130	18	221
FIT	2	8	54	6	70
Both tests	4	12	49	12	77
Total	20	79	233	36	368

### Statistical model

The model excluded the variable of the level of knowledge of CRC as it was highly correlated with having undergone preventive examinations. Considering the best AIC, we do not include sex in the model. Table 4 presents the results. The base (reference) was patients aged 45–49 years, with senior technician education, affiliated with FONASA groups C or D, with medical check-ups performed less than 1 year ago, socioeconomic level C3, residing in the RM, and reporting a good state of health.

**Table 4:** Results of the logistic regression.

Variables considered	Beta	Odds ratio	*p* value	95% CI
Constant	− 0.23	0.79	0.11	[− 0.51, 0.05]
Age				
50–54	0	1	0.98	[− 0.24, 0.23]
55–59	0.1	1.11	0.43	[− 0.15, 0.36]
60–64	0.58	1.79	< 0.01	[0.32, 0.85]
64–69	0.64	1.90	< 0.01	[0.37, 0.92]
70+	0.87	2.39	< 0.01	[0.57, 1.17]
Education				
Compulsory education	− 0.26	0.77	0.02	[− 0.47, − 0.04]
Professional	− 0.03	0.97	0.81	[− 0.26, 0.20]
Postgraduate	0.31	1.36	0.05	[0.00, 0.63]
SES				
AB	− 0.13	0.88	0.62	[− 0.66, 0.39]
C1a	0.05	1.05	0.74	[− 0.26, 0.37]
C1b	0.01	1.01	0.96	[− 0.28, 0.29]
C2	0.36	1.43	< 0.01	[0.12, 0.59]
D	0.22	1.25	0.10	[− 0.05, 0.49]
E	0.30	1.35	0.13	[− 0.09, 0.69]
Health insurance				
ISAPRE	0.52	1.68	< 0.01	[0.30, 0.74]
Armed forces	0.79	2.20	< 0.01	[0.30, 1.28]
FONASA groups A and B	− 0.17	0.84	0.12	[− 0.38, 0.04]
FONASA does not know group	− 0.20	0.82	0.21	[− 0.51, 0.11]
Other/Don't know	− 0.99	0.37	0.11	[− 2.20, 0.23]
Region				
North	− 0.23	0.79	0.02	[− 0.44, − 0.03]
South	0.02	1.02	0.87	[− 0.17, 0.20]
Last medical check-up				
Between one and three years	− 0.44	0.64	< 0.01	[− 0.64, − 0.24]
More than 3 years ago	− 1.01	0.36	< 0.01	[− 1.31, − 0.70]
I don’t remember	− 1.96	0.14	< 0.01	[− 2.43, − 1.49]
Perception of health status				
Very bad	1.51	4.53	< 0.01	[0.77, 2.25]
Bad	0.44	1.55	0.04	[0.02, 0.86]
Fair	0.05	1.05	0.55	[− 0.13, 0.24]
Excellent	− 0.01	0.99	0.96	[− 0.28, 0.27]

Base case: from 45 to 49 years old, with senior technician education, SES C3, health insurance FONASA C + D, with residence in the RM, with last medical check-up performed less than a year ago and reporting good health. All the variables considered are categorical.

Age was associated with a different likelihood of undergoing CRC tests, with an increased probability for older patient groups. Patients with postgraduate degrees were 36% more likely to undergo CRC tests, and those with compulsory education had a 23% lower probability of undergoing CRC tests than the base case.

Patients belonging to SES C2 had a higher probability of undergoing CRC tests. Members of the Armed Forces’ health insurance and ISAPRE patients were also more likely to undergo these tests, with an increased probability of 120% and 68% respectively.

Patients from the north were 21% less likely to undergo CRC tests. The frequency of medical check-ups was significant for all categories analyzed, with a higher probability of undergoing CRC tests the more recent the last medical check-up.

Finally, patients who reported having bad and very bad health were more likely to obtain screening and diagnostic CRC tests than those who claimed to be in good health.

We perform an alternative analysis excluding people reporting “symptoms” as the reason for undergoing CRC testing. In this way we expect to have a clearer view of the propensities of having a CRC screening rather than a diagnostic procedure. The results of this analysis are presented in the supplementary material Appendix E, Table E.2.

The result of the multinomial logistic regression model to determine the variables that explain the barriers to the performance of CRC screening or diagnostic tests is presented in the supplementary material Appendix E, Table E.3, where the reason “lack of knowledge” was used as a basis for reporting, along with the same baseline as in the previous model.

Women and people older than 70 years old were less likely to report lack of access and psychosocial factors as barriers. Also, people reporting bad health status were less likely to report psychosocial factors as barrier compared with baseline. Less frequent medical check-ups were positively associated with lack of access as a reported barrier. Patients belonging to SES D and those reporting a bad health were more likely to mention lack of access as a barrier. Lastly, patients with health insurance provided by the Armed Forces and ISAPRE and those between 50 and 64 years old were more likely to report psychosocial factors to accessing CRC tests.

## Discussion

The present study was motivated by previous research, where substantial differences were observed in the survival of patients with CRC according to the type of health insurance (even when the diagnosis and treatment are guaranteed by law), the complexity of the hospital, and the geographic location^9^. Of the many reasons that can explain these differences, one of the most important hypotheses is that patients with CRC have significant differences in the progression of the disease at the time of detection, which depends on sociodemographic factors.

Unfortunately, in Chile, there is no national cancer registry, and therefore there is not enough information on the staging of cancers at the time of diagnosis. This study seeks, through the survey of patients stratified into groups of interest, to determine if there are significant differences in the use of CRC screening and diagnostic tests, which if true would affect the level of progression of the disease at the time of detection.

As the results show, significant differences were found between users of the public and private health systems, with 83.7% and 70.4% of users having never had a CRC screening or diagnostic test, respectively. The logistic model indicates that a user of the private system is 1.68 times more likely to be screened for CRC than one in the public system.

We also found that patients with postgraduate degrees were 36% more likely, while those with compulsory education were 23% less likely to obtain CRC tests compared to those with senior technical education. In Chile, the socioeconomic level of the population and their health insurance are directly related^7^, a fact that was also found in our survey with 90% of SES AB insured in ISAPRE (47% considering AB, C1a, C1b and C2), and less than 1% of those in SES E (8% considering C3, D and E). Thus, the main results of international studies were recovered^11^ since it was verified that socioeconomic level and educational level have an impact on the propensity to obtain CRC exams.

When excluding those undergoing CRC testing due to symptoms, we have similar conclusions compared to those when considering the total sample. In this version of the model, people reporting excellent health were 36% more likely to undergo CRC screening than those reporting “good health”. Moreover, the differences in the likelihood of undergoing CRC screening tests increased for people with private insurance and postgraduate education and decreased for people having their last medical checkup more than three years ago and for those “not remembering” when they had their last medical checkup. This finding suggests that people taking care of their health are more likely to get preventive screening.

Additionally, in the group of respondents who had taken screening tests, 62% of ISAPRE participants did so for personal prevention or on the advice of their physician, compared with only 50% of the FONASA participants, as shown in Fig. 1. This difference can provide evidence that at the time of CRC diagnosis, a greater proportion of public system users present a more advanced stage of cancer, which directly affects survival rates.

No significant differences were observed in the different groups of FONASA for the probability of having a CRC screening test, the reasons why these tests were done, and the barriers. These results do not allow us to conclude that among FONASA users with lower income (groups A and B), the diagnosis of CRC is made later than among users with higher SES (groups C and D) because of a lack of preventive exams, which was one of the hypotheses presented in^9^ to explain the differences in survival by FONASA groups.

The following aspects are highlighted: (i) the factors that predispose individuals to obtain exams are age (more likely to be older), a very poor state of health, having private health insurance, and postgraduate education, (ii) the patients with the greatest barriers are those with public health insurance and those who do not have frequent health check-ups, and (iii) lack of knowledge is the most mentioned barrier.

In Chile, there is no information on the number of annual colonoscopies performed at the national level. However, we estimate that 17.3% of the population over 45 years old have had such a procedure, a much higher number than the 8.7% reported in^15^ for the year 2009–2010.

In the PRENEC program, CRC was found in 0.84% of the patients included^12^. This figure is identical to the proportion of individuals excluded from the survey due to a history of CRC (0.85%). We calculate that of the 6,243,667 Chileans over 45 years of age, approximately 52,000 probably have CRC, many of them without a diagnosis.

The increasing percentage of people having a colonoscopy could lead to future reductions in CRC incidence and mortality. However, most individuals within the target groups remained out of this spontaneous and unorganized screening procedure. In this sense, the future objective should be the introduction of a well-organized screening program at the national level. In a well-organized screening program, the number of FIT procedures should be much larger than the number of colonoscopies, as it would be used to select candidates for colonoscopies. However, it is important to note that any screening campaign or policy must entail a significant increase in the number of specialists and centers equipped to cover the potential increase in demand, since our current estimate of 136,034 procedures per year, in a country with 63 coloproctologists and 509 gastroenterologists registered in their respective associations (http://sociedadcoloproctologiachile.cl; http://sociedadgastro.cl).

In what follows we discuss the major limitations and strengths of our study. One major limitation lies in the challenges associated with survey-based data collection methods, including issues of self-reporting, over-reporting, and limitations in sample size, which can compromise the reliability of findings. Additionally, grouping categories, particularly concerning reported "symptoms," poses challenges due to the potentially unspecific nature of symptoms and the ambiguity between diagnostic and screening tests. Furthermore, the concept of prevention itself warrants clarification, as it may entail early diagnosis rather than solely screening for CRC, needing careful categorization. Moreover, the presence of associations within covariates adds complexity to data interpretation. Despite the limitations, the study offers notable strengths. It sheds light on previously unexplored territory regarding the barriers and facilitators of CRC screening adoption in Chile, filling a crucial gap in available information.

## Conclusions

To the best of our knowledge, our study is the first to explore the reasons for and barriers to performing CRC screening in Chile, a country with diminishing but still existing social, economic, and cultural inequities, and without a CRC program in place. Our results may be extrapolated to countries that do not have organized screening programs. This study provides relevant information to define a CRC prevention policy since it provides guidelines not only for those who do not undergo examinations but also for the reasons for this behavior. As the main barrier to performing CRC diagnostic tests is a lack of knowledge, information campaigns, and personalized doctor-patient conversations can be very efficient, especially among ISAPRE and Armed Forces’ patients that currently have access to this type of procedure.

Furthermore, this study emphasizes the importance of not only a robust health system and well-organized screening programs but also the willingness of individuals in the target age group to participate. This insight equips policymakers with valuable guidance on program implementation, highlighting the significance of knowledge dissemination and accessibility in fostering successful screening initiatives.

### Supplementary Information

Supplementary Information.

## Data Availability

The datasets generated and/or analyzed during the current study are available in the data repository of the University of Chile, 10.34691/UCHILE/NJZGBU.
