# Supplementary material for: Barriers to the use of tests for early detection of colorectal cancer in Chile
Source: Sci Rep. 2024 Apr 17;14:8817. doi: 10.1038/s41598-024-58920-z (PMC11021417; doi:10.1038/s41598-024-58920-z)
Supplement: Supplementary file 1 — Supplementary Information. [file 41598_2024_58920_MOESM1_ESM.docx]

Supplementary material for Barriers to the use of tests for early detection of colorectal cancer in Chile

Appendix A: Description of patient groups of the FONASA public health insurance

- Group A: no income or immigrant, family subsidy (Law 18.020). 100% coverage in the public network
- Group B: up to $400,000 CLP/month. 100% coverage in the public network and very limited access to private services with copay.
- Group C: between $400,000 and $584,000 CLP/month (with three or more dependents qualifying to group B). 100% coverage in the public network and limited access to private services with copay.
- Group D: more than $584,000 CLP/month (with three or more dependents qualifying to group C). 100% coverage in the public network and limited access to private services with copay.

It is worth noting that before 2022 groups C and D had a 10 and 20% copay in the public network. For the complete description of the public insurance system [www.fonasa.cl](http://www.fonasa.cl). 1US $ = 877.80 CLP (01/01/2024).

Appendix B: Socioeconomic characterization of the Chilean population

| People per household | AB | C1a | C1b | C2 | C3 | D | E |
| --- | --- | --- | --- | --- | --- | --- | --- |
| 1 | > $2,100 | [$1,200, $2,000] | [$662, $1,200] | [$380, $661] | [$218, $379] | [$125, $217] | < $124 |
| 2 | > $3,300 | [$1,900, $3,200] | [$1,100, $1,800] | [$617, $1,000] | [$354, $616] | [$202, $353] | < $201 |
| 3 | > $4,400 | [$2,500, $4,300] | [$1,500, $2,400] | [$819, $1,400] | [$470, $818] | [$269, $469] | < $268 |
| 4 | > $5,400 | [$3,100, $5,300] | [$1,800, $3,100] | [$1,100, $1,800] | [$575, $1,000] | [$329, $574] | < $328 |
| 5 | > $6,300 | [$3,600, $6,200] | [$2,100, $3,500] | [$1,200, $2,000] | [$672, $1,200] | [$385, $671] | < $384 |
| 6 | > $7,100 | [$4,100, $7,000] | [$2,400, $4,000] | [$1,400, $2,300] | [$763, $1,300] | [$437, $762] | < $436 |
| 7 | > $7,900 | [$4,600, $7,800] | [$2,600, $4,500] | [$1,500, $2,500] | [$850, $1,400] | [$487, $849] | < $486 |

*Table B.1: Characterization of the Chilean population, considering monthly income in thousands of pesos* [*https://www.aimchile.cl/*](https://www.aimchile.cl/)*. 1US $ = 877.80 CLP (01/01/2024).*

Appendix C: Description of the survey

In this survey, we would be grateful for information about individuals’ behavior on issues related to the prevention of colorectal cancer in the Chilean population.

We are surveying a group of people, so your answer is very important and will help us understand how much people know about how to prevent this type of cancer. We appreciate your time and dedication in answering these questions.

This survey will take approximately 8 minutes. All responses are anonymous and confidential and will only be analyzed by a team of researchers from the University of Chile.

*Please answer the following question before beginning:*

Q1: *I confirm that I am of legal age, my participation is voluntary, and I am aware that I can terminate my participation at any time.*

1. No
2. Yes

Q2: *Which health insurance do you have?*

1. Public system. FONASA Group A
2. Public system. FONASA Group B
3. Public system. FONASA Group C
4. Public system. FONASA Group D
5. Public system. FONASA does not know the group
6. ISAPRE
7. Military or Police forces insurances
8. Another type of health insurance or no health insurance.

Q3: *What is the highest level reached or the current educational level?*

1. Never attended
2. Kindergarten
3. Basic education
4. Middle education
5. Senior Technician
6. Professional
7. Postgraduate

Q4: *How would you describe your general health?*

1. Very bad
2. Bad
3. Fair
4. Good
5. Excellent

Q5: *How long ago did you have your last medical check-up? A medical check-up is understood to mean going to the doctor without having a disease, excluding pregnancy checks.*

1. Less than a year ago
2. Between 1 and 3 years ago
3. More than 3 years ago
4. I don’t remember

Q6: *Have you had or do you have cancer?*

1. Yes, colorectal cancer
2. Yes, another cancer
3. No

*Q7: Which phrase do you most identify with regarding your level of knowledge about colorectal cancer?*

1. Basic, never heard of that kind of cancer
2. Medium low, I know of its existence, but no greater detail
3. Medium high, I know the symptoms
4. High, I know the disease and how to detect it
5. Very high, I know the tests that are done for its early detection and prevention

Colorectal cancer is a type of cancer that allows early detection. One way to detect it is by performing colonoscopies or fecal occult blood tests. These examinations allow detection and complete resection of polyps at the same time and can prevent the development of cancer.

Q8: *Have you had any colorectal cancer screening?*

1. Yes, colonoscopy
2. Yes, fecal occult blood test
3. Yes, both
4. No

Start of block: Yes

Q9: *How many years ago was your last colorectal cancer screening test?*

1. Less than 5 years ago
2. Between 5 and 10 years
3. More than 10 years ago
4. I don’t remember

Q10: *What is the reason you have taken these exams? You can select more than one answer.*

1. My doctor requested it for prevention.
2. My doctor requested it for symptoms or discomfort.
3. For prevention on my own initiative, since I have a family history.
4. For prevention on my own initiative, as I have known a person with colorectal cancer.
5. For prevention on my own initiative, as I received information about colorectal cancer.
6. I have another disease for which I should take these tests.
7. Other

End of block: Yes

Start of block: No

Q11: *What is the reason you have not had these exams? You can select more than one answer*

1. Do not know where to go for exam.
2. I’m scared or dislike the exam.
3. I forget to do it.
4. I don't think I need it.
5. I don’t know about these exams.
6. I didn’t know that I had to take these tests.
7. I don't have the time.
8. These exams are expensive or I don't have access.
9. I’m not old enough yet.
10. Other

End of block: No

Appendix D: Classification of reasons for and barriers to CRC screening

Reasons are coded from Q10 of the survey. If more than one category was mentioned, we consider the reason with the lowest number:

1. Symptoms (answer 2)
2. Medical prevention (answer 1)
3. Personal prevention (answers 3, 4 and 5)
4. Other (answer 6)

Barriers are coded from Q11 of the survey. If more than one category was mentioned, we consider the barrier with the lowest number:

1. Psychosocial barriers (answers 2 and 3)
2. Lack of access (answers 1, 7 and 8)
3. Lack of knowledge or unawareness (answers 4, 5, 6 and 9)
4. Other

Appendix E: Additional statistical results

| Men | Symptoms | Medical prevention | Personal prevention | Other |
| --- | --- | --- | --- | --- |
| ISAPRE | 27,0% | 32,6% | 23,6% | 16,9% |
| FONASA | 23,9% | 35,2% | 32,4% | 8,5% |
| Armed Forces | 33,3% | 25,0% | 25,0% | 16,7% |
| TOTAL | 26,0% | 33,5% | 27,2% | 13,3% |
|  |  |  |  |  |
| Women | Symptoms | Medical prevention | Personal prevention | Other |
| ISAPRE | 34,2% | 31,5% | 26,0% | 8,2% |
| FONASA | 39,0% | 32,2% | 14,4% | 14,4% |
| Armed Forces | 25,0% | 25,0% | 50,0% | 0,0% |
| TOTAL | 36,9% | 31,8% | 19,5% | 11,8% |
|  |  |  |  |  |
|  |  |  |  |  |
| Men | Psychosocial barriers | Lack of access | Lack of knowledge | Other |
| ISAPRE | 10,3% | 22,3% | 67,4% | 0,0% |
| FONASA | 7,1% | 32,9% | 59,5% | 0,4% |
| Armed Forces | 11,8% | 11,8% | 76,5% | 0,0% |
| TOTAL | 8,1% | 29,7% | 61,9% | 0,3% |
|  |  |  |  |  |
| Women | Psychosocial barriers | Lack of access | Lack of knowledge | Other |
| ISAPRE | 10,1% | 15,2% | 74,7% |  |
| FONASA | 5,2% | 26,0% | 67,4% | 2,6% |
| Armed Forces | 20,0% | 10,0% | 70,0% |  |
| TOTAL | 6,5% | 23,3% | 69,1% | 1,1% |

Table E.1: Descriptive statistics from the survey, considering sex and type of insurance for the reasons to accessing CRC tests (symptoms, medical prevention, personal prevention and other reasons) and the barriers to not accessing CRC screening tests (psychosocial barriers, lack of access, lack of knowledge and other reasons).

| VARIABLES CONSIDERED | | Beta | Odds ratio | p value | 95% CI |
| --- | --- | --- | --- | --- | --- |
|  | Constant | -0.14 | 0.87 | 3.4e-1 | [-0.41, 0.14] |
| Age | 50-54 | -0,09 | 0,91 | 4.3e-1 | [-0.32, 0.14] |
|  | 55-59 | -0,1 | 0,9 | 4.7e-1 | [-0.36, 0.17] |
|  | 60-64 | 0,47 | 1,6 | 4.9e-4 | [0.21, 0.74] |
|  | 64-69 | 0,59 | 1,8 | 2.9e-5 | [0.31, 0.86] |
|  | 70+ | 0,81 | 2,25 | 1.1e-7 | [0.51, 1.11] |
| Education | Compulsory education | -0,3 | 0,74 | 6.9e-3 | [-0.52, -0.08] |
|  | Professional | -0,11 | 0,9 | 3.4e-1 | [-0.34, 0.12] |
|  | Postgraduate | 0,37 | 1,45 | 2.2e-2 | [0.05, 0.68] |
| SES | AB | -0,42 | 0,66 | 1.2e-1 | [-0.94, 0.11] |
|  | C1a | -0,02 | 0,98 | 9.2e-1 | [-0.33, 0.29] |
|  | C1b | -0,19 | 0,83 | 1.9e-1 | [-0.47, 0.09] |
|  | C2 | 0,26 | 1,3 | 3.0e-2 | [0.02, 0.49] |
|  | D | 0,09 | 1,09 | 5.1e-1 | [-0.18, 0.37] |
|  | E | -0,42 | 0,66 | 1.2e-1 | [-0.94, 0.11] |
| Health insurance | ISAPRE | 0,64 | 1,9 | 2.1e-8 | [0.42, 0.87] |
|  | Armed forces | 0,77 | 2,16 | 2.1e-3 | [0.28, 1.25] |
|  | FONASA groups A and B | -0,15 | 0,86 | 1.8e-1 | [-0.37, 0.07] |
|  | FONASA does not know group | 0,08 | 1,08 | 5.9e-1 | [-0.22, 0.38] |
|  | Other/Don't know | -0,6 | 0,55 | 2.8e-1 | [-1.67, 0.48] |
| Region | North | -0,16 | 0,85 | 1.3e-1 | [-0.36, 0.05] |
|  | South | 0,03 | 1,03 | 7.5e-1 | [-0.16, 0.22] |
| Last medical check-up | Between one and three years | -0,28 | 0,76 | 5.0e-3 | [-0.47, -0.08] |
|  | More than 3 years ago | -1,2 | 0,3 | 4.8e-13 | [-1.52, -0.87] |
|  | I don’t remember | -2,25 | 0,11 | 9.e-17 | [-2.77, -1.72] |
| Perception of health status | Very bad | 1.51 | 4.53 | <0.01 | [0.77, 2.25] |
|  | Bad | 0.44 | 1.55 | 0.04 | [0.02, 0.86] |
|  | Fair | 0.05 | 1.05 | 0.55 | [-0.13, 0.24] |
|  | Excellent | -0.01 | 0.99 | 0.96 | [-0.28, 0.27] |

*Table E.2: Results of the logistic regression without considering people reporting “symptoms” as the reason to undergone CRC testing. Base case: from 45 to 49 years old, with senior technician education, SES C3, health insurance FONASA C + D, with residence in the RM, with last medical check-up performed less than a year ago and reporting good health. All the variables considered are categorical.*

|  |  | Lack of Access | | | |  | Psychosocial Barriers | | | |
| --- | --- | --- | --- | --- | --- | --- | --- | --- | --- | --- |
| Variable |  | estimate | std.error | statistic | p.value |  | estimate | std.error | statistic | p.value |
|  | (Intercept) | 0,03 | 0,23 | 0,12 | 0,91 |  | -0.91 | 0.25 | -3.62 | 0.00 |
| Age | 50-54 | -0,38 | 0,19 | -2,04 | 0,04 |  | 0.42 | 0.19 | 2.23 | 0.03 |
|  | 55-59 | -0,03 | 0,21 | -0,14 | 0,89 |  | 0.42 | 0.22 | 1.97 | 0.05 |
|  | 60-64 | -0,29 | 0,24 | -1,19 | 0,23 |  | 0.66 | 0.24 | 2.80 | 0.01 |
|  | 64-69 | -0,36 | 0,25 | -1,42 | 0,16 |  | -0.33 | 0.28 | -1.18 | 0.24 |
|  | 70+ | -0,89 | 0,32 | -2,84 | 0 |  | -0.91 | 0.35 | -2.56 | 0.01 |
| Education | Compulsory education | 0,19 | 0,18 | 1,03 | 0,3 |  | 0.09 | 0.19 | 0.46 | 0.65 |
|  | Professional | 0,02 | 0,2 | 0,12 | 0,9 |  | 0.16 | 0.20 | 0.82 | 0.41 |
|  | Postgraduate | 0,01 | 0,33 | 0,04 | 0,96 |  | 0.36 | 0.30 | 1.20 | 0.23 |
| SES | AB | 0,4 | 0,52 | 0,77 | 0,44 |  | 0.07 | 0.47 | 0.16 | 0.87 |
|  | C1a | -0,35 | 0,3 | -1,17 | 0,24 |  | -0.40 | 0.30 | -1.34 | 0.18 |
|  | C1b | -0,16 | 0,26 | -0,64 | 0,52 |  | 0.29 | 0.24 | 1.20 | 0.23 |
|  | C2 | -0,01 | 0,21 | -0,03 | 0,97 |  | 0.14 | 0.21 | 0.68 | 0.50 |
|  | D | 0,69 | 0,22 | 3,15 | 0 |  | 0.80 | 0.23 | 3.44 | 0.00 |
|  | E | 0,12 | 0,31 | 0,37 | 0,71 |  | 0.65 | 0.32 | 2.03 | 0.04 |
| Health insurance | ISAPRE | -0,22 | 0,2 | -1,06 | 0,29 |  | 0.57 | 0.20 | 2.90 | 0.00 |
|  | Armed forces | -1,14 | 0,63 | -1,81 | 0,07 |  | 1.04 | 0.44 | 2.37 | 0.02 |
|  | FONASA groups A and B | -0,11 | 0,17 | -0,64 | 0,53 |  | 0.16 | 0.18 | 0.87 | 0.38 |
|  | FONASA does not know group | -0,27 | 0,24 | -1,11 | 0,27 |  | -0.50 | 0.27 | -1.81 | 0.07 |
|  | Other/Don't know | -0,07 | 0,73 | -0,1 | 0,92 |  | -0.15 | 0.75 | -0.21 | 0.84 |
| Region | North | -0,11 | 0,17 | -0,64 | 0,52 |  | -0.18 | 0.18 | -0.97 | 0.33 |
|  | South | -0,15 | 0,17 | -0,93 | 0,35 |  | 0.22 | 0.16 | 1.38 | 0.17 |
| Last medical check-up | Between one and three years | 0,47 | 0,17 | 2,69 | 0,01 |  | 0.67 | 0.17 | 3.88 | 0.00 |
|  | More than 3 years ago | 0,43 | 0,22 | 1,94 | 0,05 |  | 0.74 | 0.22 | 3.41 | 0.00 |
|  | I don’t remember | 0,5 | 0,24 | 2,07 | 0,04 |  | 0.94 | 0.24 | 3.84 | 0.00 |
| Perception of health status | Very bad | 1,96 | 0,78 | 2,5 | 0,01 |  | -12.63 | 0.00 | (*) |  |
|  | Bad | 0,75 | 0,37 | 2,03 | 0,04 |  | -1.20 | 0.58 | -2.06 | 0.04 |
|  | Fair | 0,7 | 0,15 | 4,55 | 0 |  | 0.29 | 0.16 | 1.86 | 0.06 |
|  | Excellent | -0,37 | 0,26 | -1,42 | 0,15 |  | -0.43 | 0.25 | -1.71 | 0.09 |
| SEX | Woman | -0,49 | 0,15 | -3,37 | 0 |  | -0.47 | 0.15 | -3.23 | 0.00 |

*Table E.3: Results of multinomial logistic regression. The participants surveyed were from 45 to 49 years old, with compulsory education, male, NSE C3, FONASA C + D health insurance, with residence in the RM, with last medical check-up performed less than a year ago and reporting good health. All the variables considered are categorical. (*) No respondent declaring very poor health mentioned psychosocial barriers.*
